# Evaluation of Parents’ Use of a Child Health Care Information App and Their Health Literacy: Cross-Sectional Study

**DOI:** 10.2196/48478

**Published:** 2024-04-11

**Authors:** Masahiko Sakamoto, Hirono Ishikawa, Asuka Suzuki

**Affiliations:** 1 Teikyo University Graduate School of Public Health, Tokyo, Japan

**Keywords:** health literacy, European Health Literacy Survey Questionnaire, HLS-EU-Q47, child, preschool, parent education, health care knowledge, apps, digital media, emergency room visit, mobile phone

## Abstract

**Background:**

Recently, digital media, including internet websites and smartphone apps, have become popular resources for parents in searching for child health care information. Higher health literacy among parents in obtaining adequate health care information and making proper decisions may lead to improved child health outcomes and a reduction in the burden on health care professionals. However, few studies have examined the association between the provision of child health care information apps and parents’ health literacy.

**Objective:**

This study aims to evaluate whether parents’ use of an app that provides child health care information is associated with their health care knowledge, their health literacy, and emergency room visits for their children.

**Methods:**

Participants were recruited during checkups for their 1.5-year-old children at health centers within Saku City in 2022. Parents who agreed to participate were included in this study; individuals were excluded if they were not the mother or father of the child or did not have a smartphone. Participants were asked if they had used the Oshiete-Doctor app, which was distributed by Saku City free of charge to improve the home nursing skills of parents and guardians. Sociodemographic data of parents and children, data on health care knowledge about children, data on the frequency of emergency room visits in the past 6 months, and health literacy scores (HLSs) of parents (measured with the HLS-EU-Q47 [European Health Literacy Survey Questionnaire]) were collected from participants in this cross-sectional survey. Univariable and multivariable analyses were conducted to examine the associations of app use with health care knowledge, health literacy, and emergency room visits.

**Results:**

In total, 251 respondents completed the survey (response rate: 251/267, 94%). Although the proportion of health care workers was significantly higher among app users than among non–app users (*P*=.005), no other participant attributes were significantly associated with the use of the app. The proportions of participants with higher health care knowledge and participants with higher total HLSs were significantly higher among app users than among non–app users (*P*=.001 and *P*=.003, respectively). After adjusting for potentially confounding covariates, these proportions were still significantly higher among app users than among non–app users (*P*=.02 and *P*=.007, respectively). Emergency room visits were significantly more frequent among app users than among non–app users (*P*=.007) in the univariable analysis, but the association was not significant (*P*=.07) after adjusting for sociodemographic variables.

**Conclusions:**

This study showed a significant association between parents’ use of a child health care information app and higher child health care knowledge and health literacy. The use of the app may lead to more appropriate health decisions and behaviors in children’s health care. Future studies are needed to evaluate the association between app use and emergency room visits.

## Introduction

Despite the increased health concerns during the COVID-19 pandemic, parents were discouraged from visiting medical facilities to avoid infection [1], and opportunities for classes and social gatherings to provide health care information to their children were limited. Ishikawa et al [2] reported a decline in health literacy among people during the COVID-19 pandemic.

Health literacy is the ability to access, understand, evaluate, and apply health care information, and it is related to health behaviors and outcomes [3]. Greater parental health literacy in obtaining adequate health care information and making proper decisions may lead to appropriate health care service use, which, in turn, may result in improved child health outcomes while alleviating health care professional burdens.

In recent years, mobile health (mHealth) has emerged as an effective approach to improving health literacy. Digital media, including internet websites and smartphone apps, have become popular resources for parents in searching for health care information [4]. A cross-sectional study conducted in Switzerland reported that >90% of parents with children younger than 2 years use digital media to obtain health care information regarding their children, and their main reason for doing so is having 24-hour access to such information [5]. mHealth apps are wellness programs that are available on smartphones and other mobile devices. In 2017, more than 325,000 mHealth apps became available worldwide [6]. Parents have high expectations for mHealth apps. According to a focus group study in Australia, child-rearing mothers place a high value on using web-based sources and apps to receive information and support [7].

There is great potential for using mHealth apps to obtain information, and an increasing number of initiatives use apps to provide health education [8]. A systematic review showed that IT-based interventions can result in positive health literacy outcomes [9]. For example, providing information via an app, in addition to the traditional method of providing printed booklets during pregnancy, reduces the risk of postpartum depression [10]. Further, a study in China reported that an app-based intervention that included injury prevention significantly improved the safety behaviors of parents of preschool children [11].

The inappropriate use of pediatric emergency departments is a growing concern worldwide [12,13]. In Japan, many municipalities provide free medical care for children. Consequently, many visits to pediatric emergency rooms are nonurgent. Previous studies suggested that lower parental health literacy, in addition to sociodemographic factors, is associated with increased nonurgent emergency room visits [14,15]. One of the possible benefits of improving health literacy through apps is the potential to reduce the inappropriate use of pediatric emergency departments. However, few studies have examined the association between the provision of child health care information apps and parents’ health literacy.

Since 2016, we have been conducting a project in which a health care information app for parents—Oshiete-Doctor (which translates to *Doctor, tell me*)—is being used to improve parents’ nursing skills at home. This app is free and uses numerous friendly illustrations to make information accessible to parents with low health literacy. The app is supervised by pediatricians and provides information on emergency room visits and knowledge on home care for childhood diseases. The app is expected to help parents make appropriate decisions regarding their children’s health care.

This study aimed to evaluate the associations between parents’ use of the health care information app and their health literacy, their health care knowledge, and pediatric emergency room visits for their children. We hypothesized that parents using the app would be more likely to have correct knowledge about their children’s health care, have higher health literacy, and use the pediatric emergency room less frequently when compared to parents who do not use the app.

## Methods

### Ethical Considerations

This study was approved by the Institutional Review Board of Teikyo University (approval number: 22-011-2). Participants’ selection of the item “Agree to participate” in the introductory statement of the questionnaire was considered as consent to participate in this study. The statement introduced this study and described that participation was voluntary, consent could be withdrawn at any time, data would be anonymized, and participants would be offered a ¥500 (US $3.30) Amazon gift voucher as compensation.

### Study Participants and Data Collection

Parents of 1.5-year-old children who participated in the medical checkups provided at 3 health centers in Saku City, Nagano Prefecture, between May 11 and October 5, 2022, were recruited. The principal investigator (MS, a pediatrician) explained the study to each participant, and those who agreed to participate were included in this study.

Individuals were excluded if they were not the mother or father of the child or if they did not have a smartphone. The city staff collected the questionnaires.

### Description of the App

The Oshiete-Doctor app, which was developed in 2016, is funded by Saku City and is provided free of charge to improve the home nursing skills of parents and guardians. It provides information on the following five topics: guidelines regarding hospital visits for children who are sick, explanations for child illnesses, information on vaccinations, information on childcare support groups, and disaster countermeasures for children. In Saku City, information regarding the app is distributed to all citizens when they register for birth certificates at the city office. The app is used throughout Japan and has been downloaded nearly 400,000 times.

### Measures

#### Health Literacy Score

The Japanese version of the HLS-EU-Q47 (European Health Literacy Survey Questionnaire) [16] was modified to measure parental health literacy (ie, parents’ health literacy scores [HLSs]). The original HLS-EU-Q47 was developed to measure health literacy in the general population based on a conceptual framework reflecting 4 information-processing dimensions (accessing, understanding, appraising, and applying) within 3 health domains (health care, disease prevention, and health promotion) [17], and it has been validated in a Japanese population [15]. The difficulty level of each item was rated on a 4-point Likert scale (1=very difficult; 2=difficult; 3=easy; 4=very easy), with a higher score indicating higher health literacy. In this study, we used 16 questions in the health care domain (items 1-16) by replacing “you” with “your child” to measure parental health literacy related to child health (eg “On a scale from very easy to very difficult, how easy would you say it is to find information about symptoms of illnesses that concern *your child*?”). In the health care domain, health literacy refers to the ability to access information on medical or clinical issues; understand, interpret, and evaluate medical information; make informed decisions on medical issues; and comply with medical advice [17]. We considered the questions in the health care domain to be appropriate for evaluating the competencies that users can improve by referring to the app. First, the app provides information on symptoms of children’s illnesses (question 1), treatments (question 2), and emergency procedures (question 3) to help parents access accurate information. Second, this app includes detailed instructions on emergency procedures for children, hospital visits (question 7), and medications (question 6 and question 8) to promote accurate understanding of the information. Third, by providing accurate information that is verified by a physician, the app helps parents improve their competency for evaluating medical information (question 9 and question 12). Fourth, the app introduces recommendations for visiting a doctor and managing children who are sick (question 13), as well as for emergency medical calls (question 15). As a result, the capacity to make decisions and apply medical behaviors can be improved. We included the set of questions used in this survey in Multimedia Appendix 1. The total scores for the health care domain and the subscale scores for each dimension (ie, accessing, understanding, appraising, and applying) within the health care domain were calculated. Following a previous study [18], scores of participants who answered fewer than 80% of the questions were excluded from the analysis. The index score was standardized to unified metrics, ranging from 0 to 50, by using the following formula: (mean − 1) × (50/3). The Cronbach α for the total scale was 0.886. In the analysis, participants were divided into 2 groups based on their median scores. Although binarization may result in information loss, we believed that the benefits of binarization are significant, in that it facilitates data interpretation and reduces model complexity.

#### Health Care Knowledge

Health care knowledge was assessed by using the following three statements on important and common pediatric emergencies, for which the app provides information: (1) “if your child develops a fever at night, you should take him/her to the hospital immediately, even if he/she is healthy and hydrated”; (2) “when a child is drowning, you should notice because he/she will be making noises”; and (3) “when a child vomits due to gastroenteritis, disinfection with alcohol is effective.” Responses to these statements were rated on a 4-point Likert scale (1=agree; 2=somewhat agree; 3=somewhat disagree; 4=disagree), with higher scores indicating better health care knowledge. The total scores for the three statements were calculated as the health care knowledge scores. In the analysis, health care knowledge scores were divided into high or low scores based on the median.

#### Emergency Room Visits

Respondents were asked about the number of emergency room visits that their children had within the past 6 months.

#### Use of the Oshiete-Doctor App

The use of the Oshiete-Doctor app was assessed based on their answers to the following question: “Do you use apps for childhood illnesses or well-being?” The respondents were asked to select 1 or more of the following four options: (1) “Oshiete-Doctor app”; (2) “Q-SUKE, the app developed by the Fire and Disaster Management Agency”; (3) “other apps”; and (4) “did not use an app.” Based on their responses, respondents were classified as either Oshiete-Doctor app users or Oshiete-Doctor app nonusers.

### Sociodemographic Data

We included sex, age, education status, self-rated economic status, current health care worker status, gestational weeks, birth weight, birth order, and past medical history of the children in our analysis. Education status was divided into 3 categories (high school graduate or lower, vocational school and 2-year college, and university graduate or higher) and then classified as “university graduate” or “non–university graduate.” With regard to self-rated economic status, participants reported their subjective economic status as “very good,” “good,” “fair,” “poor,” or “very poor.” Self-rated economic status was then classified as “very good - good,” “fair,” or “poor - very poor.” With regard to current health care worker status, participants who answered that they were health care workers were categorized as “yes,” whereas participants who answered that they were not health care workers were categorized as “no.” Gestation weeks were classified as either “<37 weeks” or “≥37 weeks.” Birth weights were classified as either “<2500 g” or “≥2500 g.” Birth order was divided into 4 categories (first, second, third, and fourth or more) and then classified as either “first child” or “second child or more.” With regard to the past medical history of the children, participants who answered that their children had any past medical history were categorized as “yes,” whereas participants who answered that their children did not have any past medical history were categorized as “no.”

### Data Analysis and Statistics

Continuous variables were described as summary statistics (mean and SD or median and quartiles), while categorical variables were expressed as frequencies and percentages. The participants were divided into 2 groups based on their use of the Oshiete-Doctor app, and sociodemographic characteristics were compared by using the chi-square test or 2-tailed *t* test.

We also conducted univariable analyses (chi-square test or *t* test) and multivariable analyses (multiple logistic regression analyses) to examine the association between participant demographics and HLSs, as well as the association between emergency room visits and health care knowledge scores.

In the multivariable analysis, we included attributes of parents (sex, age, education, self-rated economic status, and health care professional status) and children (sex, birth order, and past medical history) as variables.

We included these variables because previous studies have shown that they may influence the association between pediatric emergency visits and parental health literacy [19-21]. A *P* value of <.05 was considered statistically significant. Stata 17 (StataCorp LLC) was used to analyze the data.

## Results

### Respondents’ Characteristics

**Figure 1.**
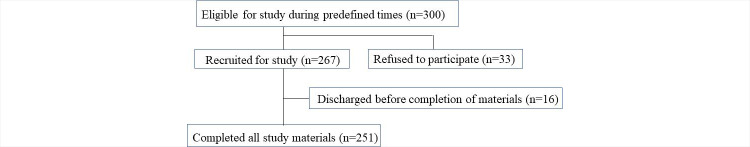
Study participant flow diagram.

A flowchart of the survey is shown in Figure 1. A total of 251 respondents completed the survey (collection rate: 267/300, 89%; response rate: 251/267, 94%). Of them, 109 (43.4%) used the app. As shown in Table 1, 90.8% (228/251) of the respondents were female, 37.1% (93/251) had a university degree or higher, and 46.2% (116/251) of their children were firstborn. Overall, 20.3% (51/251) of the respondents were health care workers, and this proportion was significantly higher among app users than among non–app users (*P*=.005). No other participant or child attributes were significantly associated with app use.

**Table 1.** Characteristics of app users and app nonusers.

Characteristics		App users (n=109)	App nonusers (n=142)	All participants (N=251)	*P* value^a^
**Sex, n (%)**					.08
	Female	104 (95.4)	124 (87.3)	228 (90.8)	
	Male	5 (4.6)	15 (10.6)	20 (8)	
	Missing data	0 (0)	3 (2.1)	3 (1.2)	
Age (y), mean (SD)		33 (5)	33 (6)	33 (5)	.95
**Education, n (%)**					.13
	High school graduate or lower	18 (16.5)	37 (26.1)	55 (21.9)	
	Junior or technical college	44 (40.3)	48 (33.8)	92 (36.7)	
	College degree or above	45 (41.3)	48 (33.8)	93 (37.1)	
	Missing data	2 (1.8)	9 (6.3)	11 (4.4)	
**Self-rated economic status** ^b^ **, n (%)**					.14
	Poor	17 (15.6)	33 (23.2)	50 (19.9)	
	Normal	67 (61.5)	87 (61.3)	154 (61.4)	
	Good	23 (21.1)	19 (13.4)	42 (16.7)	
	Missing data	2 (1.8)	3 (2.1)	5 (2)	
**Health care worker, n (%)**					.005^c^
	No	75 (68.8)	119 (83.8)	194 (77.3)	
	Yes	31 (28.4)	20 (14.1)	51 (20.3)	
	Missing data	3 (2.8)	3 (2.1)	6 (2.4)	
**Gestation wk, n (%)**					.55
	<37	6 (5.5)	9 (6.3)	15 (6)	
	≥37	76 (69.7)	82 (57.7)	158 (62.9)	
	Missing data	27 (24.8)	51 (35.9)	78 (31.1)	
**Child’s birth weight (g), n (%)**					.84
	<2500	9 (8.3)	9 (6.3)	18 (7.2)	
	≥2500	74 (67.9)	82 (57.7)	156 (62.2)	
	Missing data	26 (23.9)	51 (35.9)	77 (30.6)	
**Child’s birth order, n (%)**					.93
	First	51 (46.8)	65 (45.8)	116 (46.2)	
	Second	38 (34.9)	48 (33.8)	86 (34.3)	
	Third	12 (11)	19 (13.4)	31 (12.4)	
	Fourth or more	4 (3.7)	4 (2.8)	8 (3.2)	
	Missing data	4 (3.7)	6 (4.2)	10 (4)	
**Child’s past medical history, n (%)**					>.99
	No	99 (90.8)	127 (89.4)	226 (90)	
	Yes	7 (6.4)	9 (6.3)	16 (6.4)	
	Missing data	3 (2.8)	6 (4.2)	9 (3.6)	

a *P* values were generated using a *t* test or chi-square test.

b The “Self-rated economic status” indicates the current financial status of the participants on a 3-point scale (“good”=3; “normal”=2; “poor”=1).The data were treated as a categorical variable.

c Significant at the *P*<.05 level.

### Association Between App Use and the Health Care Knowledge Score

Health care knowledge scores were examined in terms of app use (Table 2).

**Table 2.** Differences in the proportions of parents with higher health care knowledge scores by app use.

	Univariable analysis					Multivariable analysis	
	App users, n/N (%)		App nonusers, n/N (%)		OR^a^ (95% CI; *P* value)	Parents included in analysis, n	aOR^b^ (95% CI; *P* value)^c^
High health care knowledge score	80/109 (73.4)		74/142 (52.1)		2.5 (1.5-4.3; .001^d^)	218	2.1 (1.1-4.0; .02^d^)
High score for knowledge about night fever	99/108 (91.7)		127/141 (90.1)		1.2 (0.5-2.9; .67)	217	0.7 (0.3-2.1; .56)
High score for knowledge about drowning	100/108 (92.6)		133/141 (94.3)		0.8 (0.3-2.1; .58)	197	0.5 (0.1-1.6; .25)
High score for knowledge about alcohol disinfection	80/109 (73.4)		77/141 (54.6)		2.3 (1.3-3.9; .003^d^)	217	2.4 (1.3-4.4; .007^d^)

a OR: odds ratio.

b aOR: adjusted odds ratio.

c Attributes of caregivers (sex, age, education, economic status, and health care worker status) and children (sex, birth order, and past medical history) are adjusted in the logistic regression analysis.

d Significant at the *P*<.05 level.

In the univariable analysis, the proportion of parents with higher health care knowledge scores was significantly higher among app users than among non–app users (*P*=.001), and the proportion of parents with higher scores regarding alcohol disinfection during gastroenteritis was significantly higher among app users than among non–app users (*P*=.02).

After adjusting for parent and child characteristics in the multivariable analysis, these differences remained statistically significant (*P*=.003 and *P*=.007, respectively).

### Association Between App Use and Health Literacy

The mean HLS of the participants was 24.2 (SD 7.3). The differences in the proportions of parents with higher health literacy, as measured with health care-related subscales of the HLS-EU-Q47, by app use, are shown in Table 3.

In the univariable analysis, the proportion of parents with higher total HLSs was significantly higher among app users than among app nonusers (*P*=.03), and the proportion of parents with higher HLSs for the evaluation subscale was significantly higher among app users than among app nonusers (*P*=.03).

After adjusting for parent and child characteristics in the multivariable analysis, the difference in the proportion of parents with higher total HLSs between app users and app nonusers remained statistically significant (*P*=.008).

**Table 3.** Differences in the proportions of parents with higher health literacy, as measured with health care–related subscales of the HLS-EU-Q47^a^, by app use.

	Univariable analysis					Multivariable analysis	
	App users, n/N (%)		App nonusers, n/N (%)		OR^b^ (95% CI; *P* value)	Parents included in analysis, n	aOR^c^ (95% CI; *P* value)^d^
High total score	53/102 (52)		50/132 (37.9)		1.8 (1.1-3.0; .03^e^)	206	2.2 (1.2-4.0; .008^e^)
High access score	55/105 (52.4)		66/133 (49.6)		1.1 (0.7-1.9; .67)	209	1.1 (0.6-2.0; .76)
High understanding score	68/108 (63)		78/141 (55.3)		1.4 (0.8-2.3; .23)	216	1.3 (0.7-2.4; .34)
High evaluation score	53/108 (49.1)		49/140 (35)		1.8 (1.1-3.0; .03^e^)	215	1.8 (0.99-3.2; .056)
High application score	48/107 (44.8)		50/135 (37)		1.4 (0.8-2.3; .22)	211	1.8 (0.96-3.2; .07)

a HLS-EU-Q47: European Health Literacy Survey.

b OR: odds ratio.

c aOR: adjusted odds ratio.

d Attributes of caregivers (sex, age, education, economic status, and health care worker status) and children (sex, birth order, and past medical history) are adjusted in the logistic regression analysis.

e Significant at the *P*<.05 level.

### Association Between App Use and Emergency Room Visits

In the univariable analysis of the association between a history of emergency room visits in the past 6 months and app use, emergency room visits were significantly more frequent among app users (26/102, 25.5%) than among non–app users (15/130, 11.5%; odds ratio [OR] 2.6, 95% CI 1.3-5.3; *P*=.007). This association was not significant in the multivariable analysis (parents included in analysis: n=216; adjusted OR 1.9, 95% CI 0.94-4.0; *P*=.07), in which the total HLS (a binary variable) was added to the adjusted variables (model 1).

## Discussion

### Principal Results

This is the first study to examine the associations between the use of an app for parents that was developed in Japan and child-rearing parents’ health care knowledge, their health literacy, and emergency room visits for their children. The mean HLS of the participants was 24.2 (SD 7.3), which is similar to that in previous studies [16,22]. Recently, the internet has become an indispensable tool for gathering information, and mobile apps have played a significant role. Similarly, a greater number of maternal and child health interventions using mHealth technology have been observed in the field of maternal and child health [23], and mobile apps are used to support maternal and child health care interventions [24].

Our app provides knowledge about health care for children. A significant association was found between app use and accurate health care knowledge. Fadda et al [25] reported that their smartphone app intervention for parents increased parents’ knowledge of measles-mumps-rubella vaccination and parents’ psychological empowerment toward vaccinating their children. They demonstrated the effectiveness of interventions that use mobile devices to provide information. These findings are consistent with those of our study. However, in our study, there were no statistically significant differences between app users and app nonusers with high scores for the statement about visiting the emergency room at night if their child had a fever (*P*=.56) and the statement regarding their response to their child drowning (*P*=.25). This may be because these two statements were easy for most participants to answer correctly (correct answers for the statement about night fever: 226/249, 90.8%; correct answers for the statement about drowning: 233/249, 93.6%).

This study found a significant association between app use and parental health literacy. The measure of health literacy used in this study was designed to assess the subjective manageability of health-related tasks, focusing on both individuals and the underlying circumstances in which health-related tasks are performed [26]. The use of the Oshiete-Doctor app may lower the barriers to accessing, understanding, appraising, and applying health care information and thus result in the greater manageability of child health care.

Contrary to our expectations, we found a positive association between the use of the app and emergency room visits in the past 6 months. However, a systematic review reported an association between low parental health literacy and increased emergency room visit rates [27]. One possible reason for the contradictory result from the systematic review is that our study did not evaluate the appropriateness of emergency room visits. Although we hypothesized that the use of the app would reduce unnecessary emergency room visits, a higher emergency room visit rate may reflect more careful attention to the child and appropriate decisions based on the app. Another possibility is that the parents may have downloaded the app after experiencing an emergency room visit. In the region where this study was conducted, the app is widely known among health care professionals, who may have recommended the app to parents who visited the emergency room. Future studies are needed to evaluate the association between app use and emergency room visits.

### Limitations

There are several limitations to this study. First, the HLS-EU-Q47 has a limitation in its self-reporting formula. Therefore, the survey results may reflect the subjective manipulation of the participants and may not always correspond to the objective findings. However, it has been used for comparative studies in other countries, and there is also a Japanese version [16]; therefore, its reliability and validity have already been established, which is why it was chosen in our study. Second, this study was observational and could not rule out the influence of confounding environmental factors (except for the use of other apps) that could have contributed to the increase in parents’ health literacy. In addition, as this was a cross-sectional study, there is a possibility of causal reversal. Third, a high proportion of medical personnel was observed in this study (51/251, 20.3%). Nagano Prefecture, which includes Saku City, has more medical personnel per 100,000 people when compared to the national average. For example, the number of public health nurses per 100,000 people is 82.6, which is nearly double the national average (44.1 per 100,000 people) and makes Nagano Prefecture the region with the most public health nurses in the country [28]. Therefore, it may be difficult to apply our results to parents of 1.5-year-old children from other regions. However, this study exhibits small participant bias because it was conducted with participants of a municipal infant health examination. In Japan, health checkups are available for all infants, and the follow-up rate is very high. In addition, the collection rate was very high because of the full cooperation of the city. Therefore, the results are expected to accurately reflect the reality of parents raising infants and children in this region.

### Conclusions

This study showed a significant association between parents’ use of a child health care information app and higher health literacy and health care knowledge. The use of the app may help parents increase their knowledge and ability to manage their children’s health by lowering barriers to accessing, understanding, evaluating, and applying health information. Future studies are needed to evaluate the impact of app use on health behaviors, including emergency room visits.

## Supplementary material

10.2196/48478 Multimedia Appendix 1 Health literacy score questions in this survey (modified from the HLS-EU-Q47 [European Health Literacy Survey Questionnaire] health care domain).

## Abbreviations

HLS: health literacy score
HLS-EU-Q47: European Health Literacy Survey Questionnaire
mHealth: mobile health
OR: odds ratio
