# Supplementary material for: Evaluation of Parents’ Use of a Child Health Care Information App and Their Health Literacy: Cross-Sectional Study
Source: JMIR Pediatr Parent. 2024 Apr 11;7:e48478. doi: 10.2196/48478 (PMC11034361; doi:10.2196/48478)
Supplement: Multimedia Appendix 1 [file pediatrics-v7-e48478-s001.docx]

**Supplement: Health literacy score (HLS) questions in this survey (modified from HLS-EU-Q47 healthcare domain)**

On a scale from very easy to very difficult, how easy would you say it is to:

Q1. Find information about symptoms of illnesses that concern your child?

Q2. Find information on treatments of illnesses that concern your child?

Q3. Find out what to do in case of a medical emergency?

Q4. Find out where to get professional help when your child is ill?

Q5. Understand what your doctor says to you?

Q6. Understand the leaflets that come with medicine of your child?

Q7. Understand what to do in a medical emergency?

Q8. Understand your doctor’s or pharmacist’s instruction on how to take a prescribed medicine?

Q9. Judge how information from your doctor applies to your child?

Q10. Judge the advantages and disadvantages of different treatment options of your child?

Q11. Judge when you may need to get a second opinion from another doctor?

Q12. Judge if the information about illness in the media is reliable?

Q13. Use information the doctor gives you to make decisions about your childrens’ illness?

Q14. Follow the instructions on medication of your child?

Q15. Call an ambulance in an emergency?

Q16. Follow instructions from your doctor or pharmacist?
